# Detecting Chemical Hazards in Foods Using Microfluidic Paper-Based Analytical Devices (μPADs): The Real-World Application

**DOI:** 10.3390/mi9010032

**Published:** 2018-01-17

**Authors:** Marti Z. Hua, Shenmiao Li, Shuo Wang, Xiaonan Lu

**Affiliations:** 1 Food, Nutrition and Health Program, Faculty of Land and Food Systems, The University of British Columbia, Vancouver, BC V6T 1Z4, Canada; martihua@mail.ubc.ca (M.Z.H.); shenmiao.ivy.li@gmail.com (S.L.); 2 Tianjin Key Laboratory of Food Science and Health, School of Medicine, Nankai University, Tianjin 300071, China; wangshuo@nankai.edu.cn

**Keywords:** μPADs, chemical hazard, food safety, food contamination, sample preparation

## Abstract

Food safety remains one of the most important issues in most countries and the detection of food hazards plays a key role in the systematic approach to ensuring food safety. Rapid, easy-to-use and low-cost analytical tools are required to detect chemical hazards in foods. As a promising candidate, microfluidic paper-based analytical devices (μPADs) have been rarely applied to real food samples for testing chemical hazards, although numerous papers have been published in this field in the last decade. This review discusses the current status and concerns of the μPAD applications in the detection of chemical hazards in foods from the perspective of food scientists, mainly for an audience with a background in mechanical and chemical engineering who may have interests in exploring the potential of μPAD to address real-world food safety issues.

## 1. Introduction

Consuming safe and nutritious foods is a fundamental need and a prerequisite for maintaining physical and mental health for human beings [1]. Food safety remains one of the most important issues worldwide, partially because of the ever-increasing complexity of the food systems that exacerbate the challenges in ensuring food safety. Even for the United States, which has established one of the world’s best food safety systems, it was estimated by the Centers for Disease Control and Prevention that about 48 million people get foodborne illnesses per year, causing 128,000 hospitalizations and 3000 deaths [2]. Nevertheless, great efforts have been made to improve the systematic approach to ensuring food safety. The monitoring of the agri-food products through the entire food system chain, including the detection of potential food hazards, is regarded as a key aspect of this approach.

Food hazards refer to any agents with the potential to cause adverse health consequences to the consumers [3]. These agents can be mainly classified into three categories: physical, chemical, and biological hazards. Different from physical hazards that rarely cause major food safety issues, and biological hazards that can be mostly eliminated by sufficient thermal processing, numerous chemical hazards remain stable and toxic through food processing [4,5]. Some emerging chemical hazards can be induced by inappropriate processing, such as carcinogens in overheated oil [6] and cleaning chemical residues from food plants. The adverse health effects caused by some chemical hazards has been publicly reported, including the melamine contamination in infant formula powder causing kidney stone and deaths [7] and heavy metal residues in fish products. One of the recent outbreaks is the egg contamination by fipronil, which is a broad-spectrum insecticide actively used to control fleas. The outbreak involved numerous countries and resulted in the recall of millions of eggs and lots of egg products (e.g., cake mix) while hundreds of thousands of contaminated eggs had already been consumed [8,9,10].

The analytical approaches that can test the potential chemical hazards in foods, regulatory agencies, and the food industry heavily rely on chromatographic methods (e.g., gas chromatography, high-performance liquid chromatography), mass spectrometry, and immunoassays for accurate quantification [11]. On the other hand, the increasing demand for analytical tools that rapidly screen an enormous amount of food products, especially those imported goods of which the production is difficult to regulate, promotes a wide variety of studies in developing sensors and devices for qualitative, semi-quantitative and quantitative detection of all types of chemical hazards. With the requirement of being rapid, easy-to-use and low-cost, the microfluidic paper-based analytical device (μPAD) has drawn extensive attention as a promising candidate ever since it was introduced in 2007 [12]. 

A well-developed μPAD is typically a small piece of patterned paper with 2D or 3D structure that enables the test of a few to a few tens of litres of liquid sample of the target substances within a relatively short period of time. During the past decade, numerous papers have been published in the μPAD field [13]. Many μPAD publications aim at detecting food hazards or emphasize reporting novel fabrication methods and designs whilst including proof-of-concept applications involving detecting chemical contaminants that may present in foods. A recent review paper of the advance in μPAD for food and water analysis [14] summarized about 40 publications from the perspective of chemical engineering, the majority of analytes being chemicals. However, if one takes a closer look, only a few of them were able to validate the potential of using μPADs in detecting the target chemicals within real food samples or at least spiked food matrices. There seems to be a gap in understanding the detection of food hazards for researchers without adequate food science background, which could possibly have increased the challenges in translating the device fabrication techniques to real-world applications and eventually to final commercial products. Therefore, the current review discusses the current status and concerns of the μPAD applications in detecting chemical hazards in foods from the perspective of food scientists, mainly for an audience with a background in mechanical and chemical engineering who may have special interests in exploring the potential of using μPAD to address real-world food safety issues. Many excellent original research works [15,16,17], reviews [13,14,18,19,20,21,22,23,24,25] and book chapters [26,27,28] have thoroughly discussed the fundamentals, nature of paper, fabrication technique, fluid manipulation, sensing principles, etc., therefore these topics are not covered repeatedly herein. In addition, although self-claimed as μPADs and sometimes counted in reviews elsewhere, studies that actually rely on nitrocellulose membrane are not included in this review.

## 2. Chemical Hazards in Food Matrices

A decent understanding of the analyte is the key to start a μPAD project aimed at a specific application, even with a higher priority of mastering the property of the paper substrate [29]. In practice, the market should drive the research direction of μPAD development. Essentially, the demand for detecting specific chemicals in certain sample matrices comes first and then the fittest μPAD fabrication technique can be selected and optimized. This will be further discussed in the later sections.

Table 1 summarizes the classification of chemical hazards derived from the food safety guidance in the US and Canada [3], including examples of the source, specific substance, and representative food matrix for each (sub-)category. Based upon their origins and natures, chemical hazards can be sorted as follows: (1) natural toxins that come from mold, plant, and marine organisms (e.g., aflatoxin in moldy peanuts); (2) environmental contaminants including heavy metals and persistent organic pollutants (e.g., mercury in fish, polychlorinated biphenyls in milk); (3) unapproved food additives or abuse (e.g., Sudan dyes in paprika powder, nitrite in cured meat); (4) processing-induced chemicals (e.g., bisphenol A in infant formula); (5) pesticides (e.g., glyphosate in various types of foods); (6) veterinary drugs (e.g., clenbuterol in meat); (7) food sensitivities including allergens, intolerance, and sensitivities (e.g., nuts, lactose, glutamate); (8) biochemistry and molecular biology-related food products (e.g., genetically modified food); etc. Ranging from relatively simple inorganic ions and diverse organic compounds to macromolecules such as proteins, various types of hazards can be identified in specific foods.

Detecting chemical hazards in foods is vastly different from detecting those substances in pure organic/aqueous solutions, environmental/drinking water samples, or clinical samples. In most cases, food samples are much more complex than saliva or urine—the sample liquids used in point-of-care diagnostics on which many μPAD studies have focused. For instance, in a veterinary residue test [4], the food sample could be a piece of medium-rare beef loin steak comprised of muscle (high in proteins, raw and denatured), tallow (lipids, fat-soluble compounds), meat juice and solutes (e.g., salts, amino acid), everything added during cooking (e.g., sauces, cooking oil, spices), thousands of compounds generated in the Maillard reaction, etc. Due to the diversity of chemical hazards and foods, the interaction between the analyte and the matrix can be very complicated. Depending upon the nature of both the analyte and the matrix, strong physical and chemical interactions (e.g., physically trapped in milk emulsion, chemically bound to mono/oligosaccharides) may significantly interfere with the availability of the analyte to the detecting agent. Therefore, appropriate sample pre-treatment/preparation compatible with the selected detection method is usually required to enable the function and/or to improve the performance of such a method. In the case of using μPAD, at least the sample has to be transformed into liquid form. Typically, sample preparation involves an extraction step to transfer and/or free the analyte from the food matrix, followed by a clean-up step to remove the co-extracted interfering substances as much as possible, and ended up with a (pre-)concentration (in a few cases, dilution) step if applicable. 

Bearing all the aforementioned information in mind, the gap in between “being able to detect chemical hazards” and “being able to detect chemical hazards in foods” seems to be quite underestimated. It makes little sense to claim that a μPAD can be applied to the detection of chemical hazards in foods without presenting the detailed result of testing real food samples [30]. At least, it should be clear that “being able to detect chemical hazards in foods” means “the chemical currently contained in a food sample can be detected” rather than “the chemical can be detected in its pure solution or a drinking water sample, and it may also exist in a food sample” [30].

## 3. Current Status and Concerns in Real-World Applications

### 3.1. Current Studies

Table 2 summarizes the publications reporting the application of μPADs to testing chemical hazards in real food samples. Although many more publications were included as applications in ensuring food safety in other reviews [13,14,20] and book chapters [26], those that do not present the result of testing real food samples were ruled out. Meanwhile, those fabricating paper-PDMS hybrid devices [31], those only with a subtle link to typical μPADs [32,33], and those being misclassified [14] (e.g., glucose as food additive) were ruled out as well. Several special cases [34,35,36] will be discussed in this section.

#### 3.1.1. Detecting Pesticides

Three studies reported their μPADs for testing several pesticides based upon a common principle, namely acetylcholinesterase (AChE) inhibition [34,37,38]. In brief, some acetylcholine substrates can be hydrolyzed by the enzyme AChE and change their colors, while the presence of AChE inhibitors (e.g., organophosphate and carbamate pesticides) interferes with such an enzymatic reaction to different extents, and therefore affect the degree of color change. 

**Figure 1:**
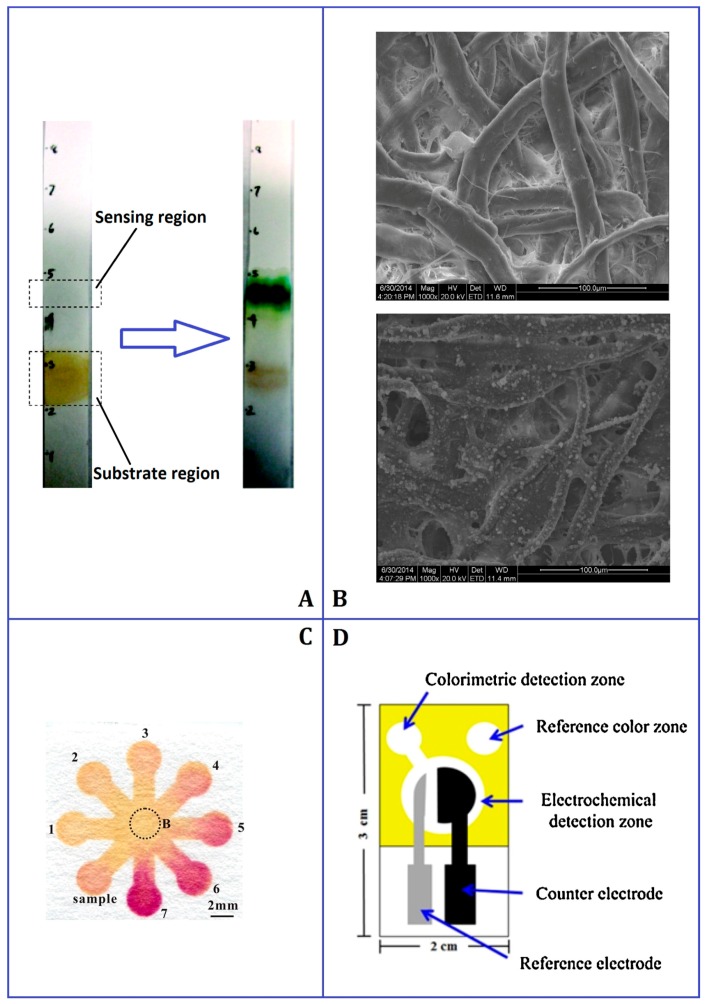
Representative μPAD applications. (**A**) Bidirectional lateral flow bioactive paper sensor. Adapted with permission from Reference [34]. Copyright 2009 American Chemical Society. (**B**) Scanning electron microscope photos of native chromatograph paper (**top**) and molecularly imprinted polymer coated chemiluminescence device. Adapted with permission from Reference [36]. (**C**) Alkylsilane self-assembling and UV/O_3_-patterning fabricated nitrite assay based on Griess reaction. Reprinted with permission from reference [39]. Copyright 2013 American Chemical Society. (**D**) Electrochemical and colorimetric dual sensor for simultaneous determination of lead, cadmium and copper. Reprinted with permission from reference [40].

In one study, Hossain et al. [34] fabricated a bidirectional lateral flow bioactive paper sensor to detect paraoxon, an organophosphate pesticide, in liquid (i.e., milk and apple juice) and on the surface of solid foods (i.e., apple and head lettuce). To fabricate the device, Whatman No. 1 paper was cut into 1 × 10 cm strips and labeled at every centimeter. The substrate region (at 3 cm) and the sensing region (at 5 cm) were functionalized through printing silica/substrate/silica and polyvinylamine/silica/AChE/silica layers of reagent inks, respectively, using a piezoelectric inkjet printer (DMP-2800, Fujifilm Dimatix Inc., Tokyo, Japan). After air drying for 1 h at room temperature, both the substrate and the enzyme were trapped within the layers of silica that fixed onto the filter paper, and the device was ready to be used. Two operation procedures were described in the study, and the bidirectional “inverted” flow system was reported to have 10-fold better limit of detection (LOD). Briefly, the 10th cm end of the strip was first dipped into the sample liquid to be tested and immediately removed once the sample reached the sensing region (5th cm), followed by 5-min incubation in the air at room temperature for better AChE inhibition. Then, the 1st cm end of the strip was immersed into ddH_2_O to bring trapped substrate (3rd cm) to the sensing region (5th cm) for the enzymatic reaction, during which the red-yellow colored substrate, indophenyl acetate, was hydrolyzed to the blue-purple indophenoxide anion (Figure 1A). Photos of the developed color were taken and analyzed by ImageJ for quantification of the pesticide concentration, referring to a built standard curve. Regarding the liquid food samples, the pesticide standard solution was mixed into the sample (incubation time not reported). No pre-treatment was performed to the milk (2%, pH 7.2), while the pH of the apple juice (pH 3.6) was adjusted to 7–8 with 1 M NaOH because the optimum pH for AChE to hydrolyze indophenyl acetate was 8.0. For solid food samples, the pesticide was sprayed onto the skin of apple and surface of head lettuce for 48 h incubation. The sample liquid was prepared by swabbing the surface of the contaminated food and dissolved in water. Such sampling method is typically used in environmental monitoring on flat, non-porous surfaces (stainless steel) for pesticide residues. In the regulations of analyzing pesticide residues in foods, the entire edible part should be included as the sampling candidate (and a minimum requirement of mass) to accurately quantify the potential hazards. This study avoided a laborious sample preparation for solid food samples, but at the same time brought the real food analysis back to the pure solution testing and decreased the value of the method validation using mass spectrometry. Nevertheless, the matrix effect of milk and apple juice was not significant. Furthermore, homogenization followed by 100× dilution may enable the detection for the solid samples in this study, since the device was able to detect as low as 0.1 nM (ca. 0.03 ppb) of paraoxon (maximum residue level = 10 ppb in Europe) in milk and apple juice. Besides, an encouraging aspect is that the strips retained >90% of their initial change in signal in a month when stored at 4 °C, which is acceptable for a commercial test strip product. 

In another study, Apilux et al. [37] developed a μPAD to detect pirimicarb, a carbamate pesticide, in lettuce and brown rice with the same principle but a different design. In this study, the AChE was directly dropped onto a punched-out Whatman No. 1 filter paper (6 mm diameter), followed by a blocking step with casein in phosphate-buffered saline (PBS) buffer, a washing step, and air dry. The methanol solution of indoxyl acetate (substrate) was spotted onto a square filter paper (10 × 10 mm). The detection procedure includes two steps as well, i.e., applying liquid sample onto the square enzyme paper for incubation and then transporting the substrate to the enzyme paper by stacking the substrate paper and adding the buffer. Regarding the sample preparation, the authors compared three methods. Immersion with 20 mM PBS buffer or 20 mM PBS buffer containing 20% methanol for 10 min only provided ca. 35% and ca. 65% recovery, respectively. However, a more laborious but efficient QuEChERS method provided excellent recovery (89–110%), although the detailed procedure was not specified. The QuEChERS method is a very popular sample preparation method in pesticide analysis for various food commodities, and the procedure and recipe can be modified to facilitate better extraction and clean-up according to the compositions. A typical QuEChERS method starts from homogenizing the food sample, followed by the addition of acetonitrile with immediate vigorous shaking. Next, MgSO_4_ (or Na_2_SO_4_) and NaCl are added, mixed and centrifuged, and then the supernatant (acetonitrile layer) will be cleaned-up by solid phase extraction. Lastly, the eluate is evaporated and reconstituted to the desired solution for further detection. In this study, appropriate sampling followed by QuEChERS sample preparation significantly improved the performance of the μPAD. The overall time for testing each sample was 45 min at least and up to 3 h. Interestingly, to achieve a better LOD (0.2 ppm), the study reported that the QuEChERS pre-treated sample liquid was applied onto the μPAD for 20 times with 5-min intervals for drying, which accounted for quite a part of the overall testing time. It was not discussed why the liquid sample was not pre-concentrated in a more time-efficient way such as the classical evaporation.

Recently, Nouanthavong et al. [38] developed a nanoceria-coated μPAD to detect methyl-paraoxon, an organophosphate pesticide, in cabbage and green mussel. The μPAD was built on Whatman No. 4 filter paper, and the 5-mm diameter circle pattern was generated by screening printing of polystyrene (25% *w*/*v* in toluene). A clear packing tape was adhered onto the back of the device to prevent leaking. In the detection zone, polyethylene glycol adjusted colloidal nanoceria solution was deposited and the device was ready to use after drying. The detection principle was partially different from the two studies summarized above although AChE hydrolysis was still the first reaction. In this study, acetylcholine was used as the substrate, and the generated choline reacted with O_2_ to produce H_2_O_2_, which then oxidized nanoceria (colorless/white) to CeO_2_ (yellow). The detection procedure was reported as adding sample liquid and incubating 15 min, followed by adding mixture (pre-incubate 15 min) of acetylcholine and choline oxidase to develop the color for 20 min. The sample preparation was similar to QuEChERS and the pre-treated sample was reconstituted in 4% methanol. Using the PAD method, the 0.2 ppm methyl-paraxon spiked into the cabbage and dried green mussel was identified to be 0.19 ± 0.02 ppm (94.8% recovery) and 0.19 ± 0.05 μg·mL^−1^ (95.1% recovery), respectively.

In all the three studies utilizing AChE inhibition, sufficient sample preparation was necessary for solid food samples, and the colorimetric method was able to quantify the target pesticides in a decent range.

Besides, Liu et al. [35,36] developed two types of chemiluminescence μPADs separately to detect dichlorvos, an organophosphate pesticide, in food samples. Both μPADs rely on the luminescence-generating oxidation of luminol by H_2_O_2_ that can be negatively affected by dichlorvos. Paper chromatography [35] and incorporated molecularly imprinted polymers [36] (Figure 1B) on Whatman Grade 3MM CHR chromatography paper were utilized to differentiate and extract dichlorvos from sample liquid, respectively. However, in spiked sample analysis (tomato, cabbage, etc.), an incorrect surface-spray-and-elute sampling and preparation were used, considering that dichlorvos is a systemic pesticide that can circulate through the plant’s tissues once absorbed.

#### 3.1.2. Detecting Inorganic Ions

Two studies [39,41] selected nitrite, a food additive possibly abused in meat products or exist in so-claimed nitrite-free products, as the analyte. Griess reaction was selected as the colorimetric principle for their μPADs in both studies. Griess reaction is a two-step diazotization reaction in which first nitrite reacts with sulphanilamide and the formed diazonium salt reacted with naphthylethylenediamine dihydrochloride to produce strong pink colored azo dye. He et al. [39] fabricated the μPAD by coupling hydrophobic octadecyltrichlorosilane (OTS) to the fibers of Whatman No. 1 filter paper, followed by UV-lithography of the OTS coating (Figure 1C). In another study, Cardoso et al. [41] used a pre-heated metal stamp to transfer paraffin wax from a waxed filter paper (JP40, JProlab, São José dos Pinhais, Brazil) to another stacked native filter paper. Both fabricated μPADs were patterned as an eight-channel-circle piece for multiple tests. Although with different major compositions, both red cubilose [39] and ham/sausage [41] were extracted with hot water due to the nature of nitrite ions with no significant matrix effect.

Chaiyo et al. developed two μPADs for detecting Cu(II) [40,42], Pb(II) and Cd(II) [40] in different food commodities. They firstly reported a colorimetric μPAD based upon the catalytic etching of silver nanoplates by thiosulfate in the presence of Cu(II) using wax printing [42]. Later, the authors incorporated the Cu(II)-detecting μPAD to another electrochemical μPAD that could detect both Pb(II) and Cd(II), forming a dual sensor [40] (Figure 1D). Regarding sample preparation, the tomato juice was simply centrifuged and filtered, while the rice was carefully treated, involving a digestion with concentrated nitric acid and concentrated perchloric acid (1:1, *v*/*v*) at 150 °C for 4 h, evaporation, titration with concentrated H_2_O_2_, and membrane filtration [42]. The procedure was slightly modified to detect all three ions together in rice and fish when electrochemistry was involved [40], including temperature change and more fine adjustment of pH (6.0). The LOD for all ions was below 10 ppb in food samples, meeting requirement of governmental regulation.

#### 3.1.3. Detecting Other Organic Compounds

Ma et al. [43] achieved the detection of clenbuterol, a veterinary drug, in milk using a paper-based enzyme-linked immunosorbent assay (ELISA). The device was fabricated by wax printing or screening patterning (with a customized mesh) on chromatography paper. A competitive ELISA with the classical horseradish peroxidase labeling format was utilized. No pre-treatment was required for milk and the LOD was 0.2 ppb. Compared to the conventional 96-well ELISA, less antibody-antigen was required and the overall detection time was reduced to about 1 h. More accurate test for other foods may require more pre-treatment to cleave the conjugate clenbuterol [48].

In addition, Liu et al. [44] and Guzman et al. [45] from the same research group developed fluorescence μPADs for detecting formaldehyde, an illegal food preserving agent, in dried goods (e.g., ginseng). With distillation (time not reported), formaldehyde in food samples was extracted and concentrated. Through Hantzsch reaction, formaldehyde reacts with ammonium and acetoacetanilide forming fluorescent dihydropyridine derivative, which can be quantified by fluorescence detecting system demonstrated in their studies.

Monosk et al. [46] applied l-glutamate, a food sensitive causing agent for some people in western countries (typically as mono-sodium glutamate), detection to instant soup sample using chromatography paper based on the dehydrogenase catalyzed color change. However, the pH of the sample had to be adjusted and the interference from l-ascorbic acid could not be avoided.

Mani et al. [47] proposed an electrochemiluminescent μPAD to screen potential genotoxic activity in the environment and foods, targeting at unspecified chemicals with genotoxicity. Whatman No. 1 filter paper was patterned by manual screen-waxing with commercially available wax paper. Different reagents (e.g., electrodes, DNA, and enzymes) were deposited in the desired structure onto two pieces of filter paper, which were then bound together using double-sided tape. In brief, when sample liquid containing genotoxic compounds applied to the μPAD, the virtual sea of DNA is damaged, which is catalyzed by cytochrome P450s (oxidative metabolic enzymes). The guanines in the damaged DNA are more accessible than intact guanine to be oxidized by Ru^III^PVP (electrically converted from Ru^II^PVP) so as to generate excited state *Ru^II^PVP which in turn decays to give electrochemiluminescence at 610 nm. In this study, over-grilled chicken was extracted with dimethyl sulfoxide and tested by this μPAD. However, a very large variation in the result was observed possibly due to the interferers extracted from the food matrix.

### 3.2. Issues and Concerns

#### 3.2.1. Challenges in Sample Preparation

It is not difficult to notice that the current studies have focused on pesticides, inorganic ions and simple organic compounds, which are small molecules without overly complex or subtle structures (Table 2). No macromolecules have been involved, such as proteins in the only two categories (i.e., food sensitivity and biochemistry-related). Precise and efficient separation of proteins from food matrix is still technically challenging, especially in the case of solid food and/or cooked food. Researchers may select compounds that can be easily extracted from foods as a start or as the model analyte in fabrication-emphasizing studies. On the contrary, many small molecules are homogeneously distributed in food sample and only weakly interact with the food matrix. Therefore, a solvent extraction should provide a decent recovery. Depending upon the detection principle, the analyte may not have to be present in the free form completely (e.g., when the sensitivity of the μPAD was very high [43] with repeatable recovery for calibration), which may reduce the technical challenges in sample preparation or be total exempted.

#### 3.2.2. Not Really Rapid

Due to the technical challenges in sample preparation, the laborious and tedious work prior to detection reveals the time issue. It is totally acceptable to describe the detection of a pesticide in the environment water sample within 10 min as a rapid test. However, applying the exactly same μPAD to a food sample, it is no more a rapid test if a two-hour sample preparation time is added into the overall turnaround. For example, in the manuals of Rapid Test Kit Evaluation Program for detecting mycotoxins in grains, the length of time defined for a “rapid test” is 30 min, including sample preparation [49]. To address this time, μPADs with a higher interference tolerance may simplify the prerequisite sample pre-treatment greatly. For instance, the clean-up step in the QuEChERS method may not be strictly required for some food commodities (e.g., milk). In this case, the analytes end up in acetonitrile (or other organic phases) containing a very small amount of water, and the co-extracted interferers vary in their composition. This leads to the next two issues regarding solvent: hydrophobic barrier and the paper substrate.

#### 3.2.3. Aqueous Solution Limited

One of the two issues is the compatibility of the sample preparation method with the μPAD, particularly the compatibility of ending liquid with the hydrophobic barrier and the paper substrate. In the aforementioned case, the ending solvent cannot be switched from acetonitrile/methanol to water or a mixture of water/alcohol due to the intrinsic reasons (e.g., free pesticide may re-conjugate to co-extract matrix when the solvent is changed from acidified acetonitrile to a water/ethanol 1:1 blend) or simply because of the concern of time and labor cost. However, some fabrication techniques may not support those organic solvents perfectly, as methanol breaking through wax-printed barrier has been observed. Dipping and efficient stamping techniques can hold methanol better as the paper substrate is more saturated with pure paraffin wax and polydimethylsiloxane (PDMS). In most studies, the fundamentals and applications were studied using an aqueous phase [16], such as colored water and PBS buffer. The wicking of organic solvents in different conditions (e.g., significantly higher evaporating effect, different solutes) may have to be further studied to promote more applications in food analysis. Special attention should be paid to acetonitrile and methanol, especially acetonitrile, since these two solvents could be the most frequently used ones in the extraction step. 

#### 3.2.4. Limited Understanding of Interactions between Paper-Analyte/Interference

The other one of the two issues is related to the limited understanding of the interaction among all participants within a wicking system of the μPAD. For those designs involving liquid wicking within the paper substrate, the interactions between analyte/interferer and paper/barrier are observed, predicted, but not well understood. A direct question regarding the interaction between the analyte and the paper is that how a large percent of the analyte can finally reach the designed sensing zone. Ota et al. [17] quantitatively evaluated the transportation of the analyte in μPAD (Advantec No. 5C filter paper patterned by wax-printing) using Ni^2+^ as a model metal cation and bovine serum albumin (BSA) as a model protein, which provided insightful conclusion for the design of a μPAD. Different regions of a protein molecule interact with the cellulose fiber (mainly the hydrophilic hydroxyl groups) and other components (e.g., lignin) to different extents, and the interaction may change with the composition of the solvent system and the resulted change in protein folding. Moreover, the working reagent conjugated to the analyte could also be retarded by the hydrophobic barrier at the interface and thus cause the variation in the transportation of the analyte [50]. Regarding the interaction between the interferers in the sample fluid and paper substrate, the original function of the chromatography paper cannot be ignored. Such separation ability based upon polarity, along with the particle trapping ability of the space among randomly oriented fibers, can be helpful when dealing with extracted but non-cleaned samples. Particularly, all these interactions within the system of acetonitrile (with up to 10% water) and methanol (containing 0–50% water) should be further investigated as they are quite common in food sample preparation.

#### 3.2.5. Market Orientation from the Beginning

Although there seems to be a long way to go from the design of a μPAD prototype to the large-scale manufacturing of a final commercial product, it is necessary to keep the target market niche in mind from the very beginning of a study. One should not only consider the nature of the target chemical hazard and possible food matrix but also the specific needs of the end users, so as to clarify the plan for the research emphasis. For an ideal design, the “ASSURED” criteria (i.e., Affordable, Sensitive, Specific, User-friendly, Rapid and robust, Equipment-free, Deliver to the users who need them) of point-of-care diagnostic devices with resource-limited settings can be introduced and compared [51]. 

Agri-food business generally has low unit value and profit, while strict regulations have to be met as the food quality and safety is directly related to consumer health. The most important benefit that a μPAD can bring to the food industry is the affordability. Essentially, the overall cost per test is what a business cares about most, and covers most of the aforementioned criteria. Different from the estimated cost per μPAD mentioned in the publications, many other factors affect the calculation to a great extent. To start with, the cost of the required equipment per test is a concern, related to the initial investment, maintenance, and the actual total test can be done on the equipment (i.e., the frequency of use, the designed time in warranty, and the time before a required update). Thus, “equipment-free” for a food plant is perhaps more a matter of cost rather than of convenience. Then, the labor cost per test, involving the overall turnaround per test and the operation capacity per person, is another factor to include. Considering the huge number of fresh produce and food products, especially imported goods that have a higher risk of chemical contamination [52], using μPAD as a fast screening method in a high-throughput manner is preferred, even though the sensitivity, accuracy and precision have to be compromised to some extent. In turn, the false positive/negative rate largely decides the extra cost of validation by typically sending out samples with questionable result to external analytical services at high prices. In the worst scenario, the failure of the μPAD may result in a costly recall and huge loss of brand value. In brief, these real-world considerations in the food industry may question the “low-cost” tag of μPADs.

“Equipment-free” and “user-friendly” become increasingly important for the applications targeting at the consumer’s level. For example, a home-use food allergen testing μPAD will for sure find its market niche if it requires no equipment (at most a smartphone), very simple or no sample preparation, and an easy-to-interpret indicator. Sometimes, even though the μPAD can be expensive, consumers may still be willing to purchase due to very serious consequences that might result from food allergy. For governmental inspectors who have to do on-site testing at farms, slaughterhouses and food plants may prefer portable readers with recording functions to obtain relatively accurate results and to reduce sampler’s bias. Therefore, the corresponding μPAD product does not have to be equipment-free, even though the test is just qualitative or semi-quantitative and can be interpreted by the naked eyes. If the application targets on the completely banned chemicals (e.g., clenbuterol), one may not have to make too much effort on the linearity of working range of the μPAD, but need to emphasize the specificity and LOD. In brief, considering the real-world scenario of a specific application of μPAD in the detection of chemical hazards in food commodities from the very start of a μPAD project could be very helpful, because it may clarify the research emphasis and reduce the developing time period for researchers of both chemical engineering and food science.

## 4. Conclusions and Future Opportunity

Although the μPAD is still in its infancy stage [26] and limited studies have been reported on the application of detecting chemical hazards in foods, more designs that are applicable to real food samples would emerge as the μPAD gains maturity and the market of food testing is more recognized. The potential of μPAD has not been explored too much in terms of (programmed) on-paper sample preparation (e.g., paper centrifuge [53]), on-paper pre-concentration, and recognition with highly specific agents (e.g., aptamers [31] and molecularly imprinted polymers [54]). Although it was suggested that current technology should be combined with the conventional PDMS-based microfluidic systems as a solution to a “sample-in-read-out” design [26], the regulations in sample size for different food commodities should also be taken into consideration. Further improvement in reducing the variation to achieve a better repeatability and reproducibility will significantly lower the overall cost and attract attention from the food industry. With more advances in the development of μPAD for real-world applications, competitive μPAD products will eventually come to the market and resource-limited regions where food safety needs to be improved urgently.

## Figures and Tables

**Table 1:** Chemical hazards in food matrices.

Category	Sub-Category	Source Example	Substance Example	Food Matrix
Natural toxins	Mycotoxin	Mould	Aflatoxin	Peanut
	Plant toxin	Plant in response to stress	Glycoalkaloids	Potato tuber
	Marine toxin	Fish decomposing	Histamine	Tuna
		Bioaccumulation from algae	Saxitoxin	Clam
Environmental contaminants	Inorganic, heavy metals	Industrial manufacture, mining, pesticide degradation	Arsenic, lead, mercury	Seafood
	Persistent organic pollutants		Polychlorinated biphenyl	Fish, milk
Unapproved food additives	-	Adulteration, importation	Sudan dye	Paprika
Processing-induced chemicals	-	Surfactant, antimicrobial, undesired reaction, migrate from container	Nitrosamines, melamine, bisphenol A	Processed foods
Pesticides/agricultural product	Herbicide, insecticide, fungicide	Agricultural practice, sanitation misconduct	Azoxystrobin	Peach
Veterinary drugs		Animal disease control	Clenbuterol	Meat
Food sensitivity	Food allergens	Cross contamination, improper labeling	Peanut, milk, fish, gluten	Various
	Food intolerances		Lactose	
	Chemical sensitivity		Monosodium glutamate	
Biochemistry-related	-	Genetically modified food, cross contamination, improper labeling, adulteration	Adulterant	Meat, flour, corn
Novel foods and others	-	New formulation or processing procedure	DNA-damaged ingredients, drugs	Various

**Table 2:** Summary of microfluidic paper-based analytical device (μPAD) applications in detecting chemical hazards in food matrices.

Analyte	Food Matrix	Sample Preparation	Liquid Phase	Principle and Format	Paper Type	Fabrication Method	Barrier Material	Note	Ref.
Paraoxon (organophosphate pesticide)	Milk, apple juice, head lettuce, apple	Adjust apple juice pH, swab surface of lettuce and apple into water	Aqueous	Acetylcholinesterase (AChE) inhibition, colorimetric bidirectional lateral flow strip	Whatman No. 1	Paper-cutting, inkjet-printing of reagents	-	Silica assisted reagent trapping, sampling method issue	[34]
Pirimicarb (carbamate pesticide)	Lettuce, brown rice	PBS buffer extraction (10 min) or QuEChERS method	Aqueous, acetonitrile	AChE inhibition, colorimetric	Whatman No. 1	Cutting	-	Sample preparation details not reported	[37]
Methyl-paraoxon (organophosphate pesticide)	Cabbage, green mussel	QuEChERS method	Aqueous (4% methanol)	AChE inhibition, colorimetric	Whatman No. 4	Polymer screen-printing method	Polystyrene	Nanoceria (CeO_2_) coated μPAD	[38]
Dichlorvos (organophosphate pesticide)	Cucumber, tomato, cabbage	Water eluting & filtration	Aqueous	Chemiluminescence, lateral flow	Whatman Grade 3MM CHR chromatography paper	Cutting, home-made reagent dispensing equipment	-	Incorrect sampling	[35]
	Tomato skin, cabbage leaf			Chemiluminescence, molecularly imprinted polymers					[36]
Nitrite	Red cubilose	75 °C water extraction 5 min, centrifugation 30 min	Aqueous	Griess-color nitrite assay	Whatman No. 1	UV-lithography	Octadecyltrichlorosilane	Sample preparation details not specified	[39]
Nitrite	Ham, sausage	100 °C water extraction (1 h)	Aqueous	Griess-color nitrite assay	JProlab JP40 filter paper	Stamping	Paraffin	-	[41]
Cu(II)	Tomato juice, rice	Centrifuge & filtration (tomato juice), 4 h acid digestion & oxidation (rice)	Aqueous	Catalytic etching of silver nanoplates by thiosulfate in presence of Cu^2+^, colorimetric	Whatman No. 1	Wax printing	Xerox Color Qube printing wax	-	[42]
Cu(II), Pb(II), Cd(II)	Rice, fish	4 h acid digestion, pH adjustment, filtration	Aqueous	Electrochemistry for Pb(II), Cd(II)	Whatman No. 1	Wax printing (pattern), screening-printing (electrochemical ink)	Xerox Color Qube printing wax	Cu(II)detection [42] incorporated	[40]
Clenbuterol (veterinary drug)	Milk	No	Aqueous	Competitive ELISA, HRP labeled	Chromatography paper	Wax printing, screening-patterning	Xerox Color Qube printing wax, paraffin	-	[43]
Formaldehyde (illegal preservative)	Dried goods	Micro-distillation	Aqueous	Hantzsch reaction, fluorescent formaldehyde-Acetoacetanilide complex	Advantec No. 1	Wax printing	Xerox Color Qube printing wax	-	[44,45]
l-glutamate	Instant soup	No	Aqueous	Dehydrogenase catalyzed color change	Chromatography paper	No pattern	-	-	[46]
“*Genotoxic activity* of pollutants”	Grilled chicken	Dimethyl sulfoxide (DMSO) extraction	DMSO	Electrochemiluminescence	Whatman No. 1	Screening-printing	Wax from commercial wax paper	Large variation, analyte not specified	[47]
